# Gibberellins as a novel mutagen for inducing 2n gametes in plants

**DOI:** 10.3389/fpls.2022.1110027

**Published:** 2023-01-11

**Authors:** Yifan Zhao, Bo Kong, Phuong Uyen Do, Liang Li, Jiahua Du, Lexun Ma, Yaru Sang, Jian Wu, Qing Zhou, Xuetong Cheng, Xiangyang Kang, Pingdong Zhang

**Affiliations:** ^1^ National Engineering Research Center of Tree Breeding and Ecological Restoration, Beijing Forestry University, Beijing, China; ^2^ Key Laboratory of Genetics and Breeding in Forest Trees and Ornamental Plants, Ministry of Education, Beijing Forestry University, Beijing, China; ^3^ College of Biological Sciences and Technology, Beijing Forestry University, Beijing, China

**Keywords:** gibberellic acid, tubulin immunolocalization, cytokinesis, 2n pollen, triploid

## Abstract

The plant hormone gibberellin (GA) regulates many physiological processes, such as cell differentiation, cell elongation, seed germination, and the response to abiotic stress. Here, we found that injecting male flower buds with exogenous gibberellic acid (GA_3_) caused defects in meiotic cytokinesis by interfering with radial microtubule array formation resulting in meiotic restitution and 2n pollen production in *Populus*. A protocol for inducing 2n pollen in *Populus* with GA_3_ was established by investigating the effects of the dominant meiotic stage, GA_3_ concentration, and injection time. The dominant meiotic stage (F = 41.882, *P* < 0.001) and GA_3_ injection time (F = 172.466, *P* < 0.001) had significant effects on the frequency of induced 2n pollen. However, the GA_3_ concentration (F = 1.391, *P* = 0.253) did not have a significant effect on the frequency of induced 2n pollen. The highest frequency of GA_3_-induced 2n pollen (21.37%) was observed when the dominant meiotic stage of the pollen mother cells was prophase II and seven injections of 10 μM GA_3_ were given. Eighteen triploids were generated from GA_3_-induced 2n pollen. Thus, GA_3_ can be exploited as a novel mutagen to induce flowering plants to generate diploid male gametes. Our findings provide some new insight into the function of GAs in plants.

## Introduction

*Populus* is a model woody plant perennial system. In this system, after two rounds of meiosis, pollen mother cells (PMCs) form a tetrad containing four haploid interphase nuclei. Then cytokinesis takes place, four unicellular haploid pollen grains are produced. Subsequently, a bicellulate pollen with a generative cell and a vegetative nucleus comes into being when the unicellular haploid pollen grains undergo rounds of mitosis. However, if a physical or chemical mutagen is present, such as colchicine or dinitroanilines (e.g., oryzalin and trifluralin), meiosis is inhibited and 2n pollen is produced (Dhooghe et al., 2009).

Mutagenic agents for inducing 2n gametes in plants include chemical mutagens, such as colchicine, oryzalin, and trifluralin. Colchicine is one of the most effective chemical mutagens and is widely used to induce 2n gametes and generate triploid plants (Li et al., 2008; Li et al., 2014; Zhou et al., 2020). Other mutagenic agents include physical mutagens, such as high-temperature exposure, cold stress, ultraviolet rays, and γ-rays (Li et al., 2001; Pécrix et al., 2011; Lu et al., 2013; Li et al., 2017). High-temperature exposure is often applied to induce 2n gametes during meiosis of meiocytes or development of the embryo sac in some woody plants, and a large number of triploids are created (Wang et al., 2012; Li et al., 2017). For example, Lu et al. (2013) reported inducing 2n female gametes during megasporogenesis and the development of the embryo sac in *Populus adenopoda* Maxim using high-temperature exposure, and 63 triploids were produced. 56.33% of 2n pollen was produced *via* high-temperature exposure during microsporogenesis in *Populus canescens* (Ait.) Smith, and forty-two triploids were generated (Tian et al., 2018; Zhou et al., 2022).

Gibberellins (GAs), one type of endogenous plant hormones, are involved in many processes of plant growth and development, such as seed germination, leaf expansion, trichome formation, pollen maturation, and floral transition (Debeaujon and Koornneef, 2000; Davière and Achard, 2013; Claeys et al., 2014). For example, GA takes part in the regulation of floral morphogenesis and reproductive system growth (Fleet and Sun, 2005). GA positively modulates the stamen development and petals in Arabidopsis through promoting cell elongation and also modulates microsporogenesis and pollen formation (Cheng et al., 2004; Plackett et al., 2014). In some plant species, GA signaling controls tapetal cell development and programmed cell death, which is necessary for the growth and maturation of developing microspores. In addition, GA operates as a signaling molecule on exposure to abiotic stress caused by either changes in the biosynthesis of GA or its downstream signaling pathway. Liu et al. (2017) reported that exogenous treatment of flowering Arabidopsis (*Arabidopsis thaliana*) plants with 100 μM GA causes defects in male meiotic cytokinesis leading to meiotic restitution and a lower frequency of diploid pollen produced. However, as < 5% of 2n pollen was induced, no triploids were produced.

In this study, we found that an exogenous gibberellic acid (GA_3_) injection of male *Populus* flower buds caused defects in the radial microtubule arrays (RMAs) during male meiotic cytokinesis resulting in meiotic restitution and 2n pollen production. A protocol for inducing 2n pollen in *Populus* using exogenous GA_3_ was established. The highest frequency of GA_3_-induced 2n pollen (21.37%) was observed when the dominant meiotic stage of the pollen mother cells (PMCs) was prophase II, and seven injections of 10 μM GA_3_ were given. Eighteen triploids derived from GA_3_-induced 2n pollen were generated. Therefore, exogenous GA is a novel type of mutagen to induce flowering plants to generate diploid male gametes. Our findings provide new insight into the functions of GAs in plants.

## Materials and methods

### Plant materials

Male floral branches of *P. bolleana* (2n = 2x = 38) were sampled from Inner Mongolia Agricultural University. Male floral branches of *P. canescens* (2n = 2x = 38) were collected from a natural population in Aletai, Xinjiang Uygur Autonomous Region. Female floral branches of *P. alba × P. glandulosa* (2n = 2x = 38) and male branches of *P. tomentosa* clone 5088 (2n = 2x = 38) were collected from Guan County, Shandong Province. All floral branches were cultured in tap water in a greenhouse (10–20°C) at Beijing Forestry University to force floral development. No extra nutrition was supplied to the tap water.

### Cytology

After the male floral branches had been cultured, two to three flower buds were collected randomly every 2 to 4 hours intervals and fixed in Carnoy’s solution until the tetrads emerged. After the fixed buds were stored at 4°C for 24 h, the anthers were dissected from the buds for the meiotic analysis using forceps and were crushed in a droplet of aceto–carmine solution (2%) on a microscopic slide. Photomicrographs of developing PMCs were taken under a light microscope (model BX51; Olympus, Tokyo, Japan) with a CCD camera (model DP70; Olympus). About 200–300 PMCs were observed in each sample to determine the dominant meiotic stages.

The male meiotic products were stained by aniline blue solution (0.1% [m/v] in 0.033% K_3_PO_4_ [m/v]) according to the method reported by Liu et al. (2017). We examined and photographed the samples using a Leica TCS-SP8 confocal laser scanning microscope.

### Tubulin immunolocalization

When the PMCs initiated meiosis, the *P. bolleana* anthers at different meiotic stages were sampled and fixed in 4% paraformaldehyde for 45 min. The tubulin-α immunolocalization was conducted according to the methods described by Zhang and Kang (2013). The anthers were extracted from fresh flower buds and fixed in 4% (v/v) paraformaldehyde for 45 min. The fixed anthers were washed three times in freshly prepared PEM buffer (50 mM PIPES, 5 mM EGTA, and 1 mM MgSO4; pH = 6.8) for 5 min. After being permeabilized with 10% (v/v) dimethylsulfoxide (DMSO) for 15 min, the treated anthers were then extracted in 1% (v/v) Triton X-100 for 15 min. After that the samples were washed three times with PEM buffer and phosphate-buffered saline (PBS; 137 mM NaCl, 8 mM Na_2_HPO4, 2.7 mM KCl, 1.5 mM KH_2_PO4; pH = 7.4), respectively. The anthers were dissected using forceps to free the meiocytes and tetrads. The cells were released on slides coated with 0.1% (w/v) poly-L-lysine and then labeled with an anti-α-tubulin antibody (T9026; Sigma, St. Louis, MO, USA) diluted 1:200 in PBS. The samples were incubated at 37°C for 2 h followed by three washes in PBS. After the hybridization with the primary antibody, all samples were then labeled with a 1:50 (v/v) dilution of fluorescein isothiocyanate (FITC) conjugated anti-mouse immunoglobulin G antibody (F5262; Sigma St. Louis, MO, USA), and incubated for 1 h at 37°C in a humid chamber. Finally, the slides were washed three times for 5 minutes with PBS and then were counterstained with 20 μl of 4’,6-diamidino-2-phenylindole (DAPI). The Leica TCS-SP8 confocal laser scanning microscope was used to examine and photograph the prepared samples.

### 2n pollen induction by GA_3_ injection

When the dominant meiotic stages of the PMCs were diakinesis, metaphase I, anaphase I, prophase II, metaphase II, and anaphase II, 10, 30, or 50 μmol/L GA_3_ solution was injected into the flower buds with a needle for 3, 5,7 times. All meiotic stages were injected with different concentrations of GA_3_ for different times. The male flower buds given 5 injections with distilled water served as the control groups. After each treatment was completed, all of the treated male flower branches were hydroponically cultured until pollen was released from the anthers.

When anthers matured, five catkins (flower spikes) were randomly sampled to determinate the frequency of induced 2n pollen in the control and each treatment group, respectively. After pollen collection, the pollen samples were stored in glass bottles containing allochronic silica gel. The estimation of the frequency of induced 2n pollen was conducted according to the method reported by Zhang and Kang (2013). When the diameter of the pollen grain was > 1.28 times as large as the average diameter of the pollen grain of the control group, the enlarged pollen grain was regarded as 2n pollen. The diameters of 300–400 pollen grains were measured per sample. The estimated frequency of 2n pollen was equal to the ratio of the number of 2n pollen to the total number of calculated pollen grains.

### Triploid production by crossing GA_3_-induced 2n pollen

The stigmas of female *P. alba* × *P. glandulosa* buds were pollinated with a relatively high frequency of GA_3_-induced 2n pollen. After pollination, the female flower branches were continuously cultured in the greenhouse until seeds matured. The seeds were collected and sown in 54 × 28 × 10 cm nutrient plates at a depth of 5 cm to promote growth. Surviving seedlings with a height of approximately 25 cm were transplanted into a field.

### Ploidy analysis by flow cytometry and counting the somatic chromosomes

Flow cytometry was used to detect the ploidy level of offsprings according to the method documented by Galbraith et al. (1983). A 55 mm Petri dish with 1 ml modified Galbraith’s buffer (0.2 mM Tris-HCl, 45 mM MgCl_2_, 30 mM sodium citrate, 20 mM 4-morpholinepropane sulfonate, 1% (v/v) Triton X-100, pH 7.0) was filled with approximately 0.5 g of chopped young leaves, and filtered through 40 μm nylon mesh. Then, 50 μl of 4′, 6-diamidino-2-phenylindole (10 mg/ml) was added to stain the nuclei for 5 min. Three samples were taken from each plant, and each sample contained at least 2,000 nuclei. A leaf sample from a known diploid plant of 84K poplar (2n = 2x = 38) was employed as an external standard. The C-value of *Populus* is 0.46 pg (Kew C-value database), which was the standard peak that appeared at about channel 50 of relative fluorescent intensity.

Chromosome counting of chromosome numbers was conducted for putative triploids. Stem tips from the offspring were cut and pretreated with *para*-dichlorobenzene solution for 3 h at room temperature. Then, the stem tips were washed in water and fixed in fresh Carnoy ‘s solution (ethanol:acetic acid, 3:1) for at least 24 h at 4°C. The fixed stem tips were hydrolyzed for 15 min at room temperature in a solution of 38% HCl: ethanol (1:1), followed by a 10-min wash in distilled water. The hydrolyzed samples were stained by Carbol fuchsin, crushed with a cover slip, and photographed at 100 X oil lensunder an Olympus BX51 microscope.

### Statistical analysis

The frequency of GA_3_-induced 2n pollen was analyzed using a GLM, which revealed the differences among meiotic stages, GA_3_ concentrations, and injection times. Before analysis of variance, a transformation (1/p) for the frequency of induced 2n pollen data was conducted due to the heterogeneity of the variance. All statistical analyses were conducted with SPSS software (SPSS for Windows, Version18.0, SPSS Inc., Chicago, IL, USA). A *P*-value < 0.05 was considered significant.

## Results

### Meiosis of PMCs in *Populus bolleana*

Meiotic analysis of PMCs can be used to guide the doubling of gamete chromosomes by a particular physical or chemical mutagen. The meiotic process of PMCs was consecutive and asynchronous in *P. bolleana* (**Table 1**) and about 4 days were needed to complete meiosis of PMCs. Owing to the asynchronous development of PMCs, each male bud contained more than one meiotic stage, similar to other *Populus* species.

**Table 1:** Meiotic process of PMCs in *P. bolleana*.

Meiotic stage of PMCs	Hours after culture (hr)											
	96	110	124	136	144	152	160	168	174	180	186	192
Leptotene	65.5	28.5	9.0	3.5								
Zygotene	25.5	51.5	36.5	14.5	5.0							
Pachytene	9.0	16.5	40.0	27.5	18.0	12.5						
Diplotene		3.5	14.5	47.5	22.0	14.0						
Diakinesis				7.0	46.0	20.5						
Metaphase I					9.0	27.0	8.5	6.0				
Anaphase I						10.0	22.5	8.5				
Telophase I						12.0	37.5	13.0	5.5	2.0		
Prophase II						4.0	21.0	34.0	28.0	11.0	5.5	0.5
Metaphase II							10.5	30.5	37.0	25.5	6.5	2.0
Anaphase II								8.0	21.0	39.0	10.5	2.5
Telophase II									8.5	19.5	50.5	14.0
Tetrad										3.0	27.0	81.0

**Figure 1:**
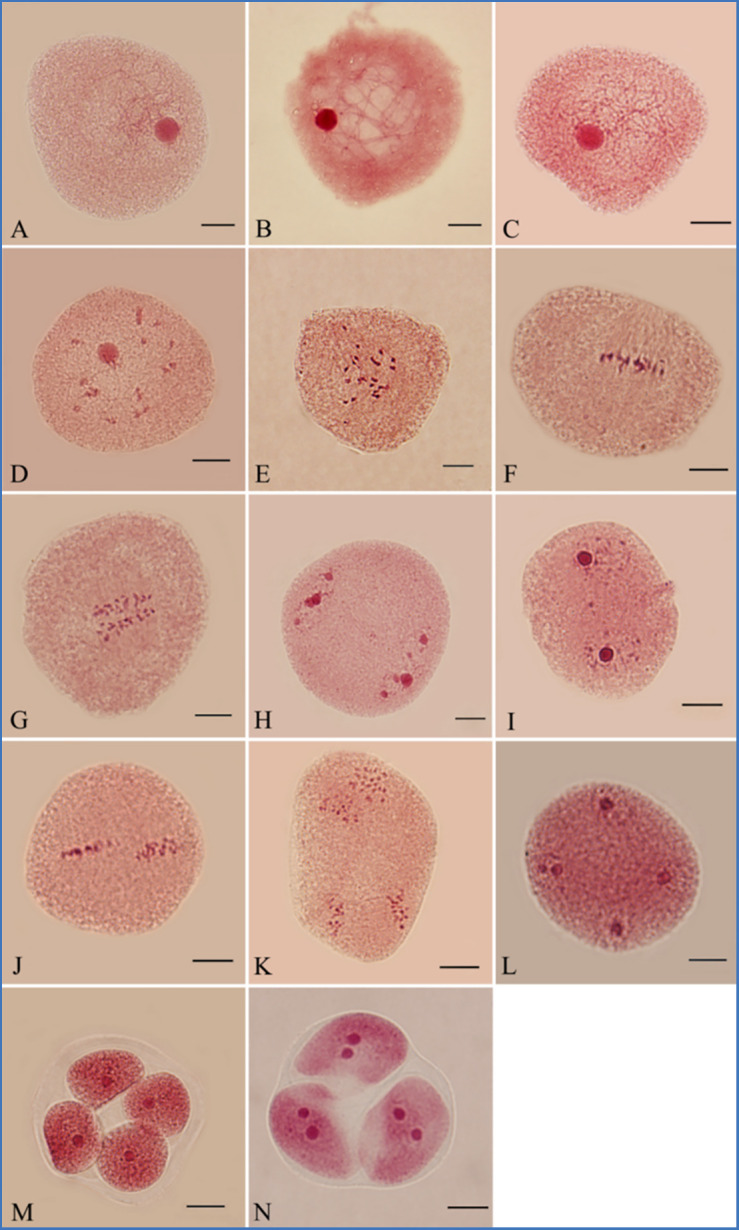
Meiosis of PMCs in *P. bolleana*. **(A)** Leptotene. **(B)** Zygotene. **(C)** Pachytene. **(D)** Diplotene. **(E)** Diakinesis. **(F)** Metaphase I. **(G)** Anaphase I. **(H)** Telophase I. **(I)** Prophase II. **(J)** Metaphase II. **(K)** Anaphase II. **(L)** Telophase II. **(M)** Tetrad. **(N)** Triad. Bars, 10 μm.

PMCs underwent meiosis initially after 96 h of greenhouse culture. Most PMCs were at leptotene (65.5%; **Figure 1A**). The dominant meiotic stage changed from zygotene to diakinesis after 110–144 h of culture (**Figures 1B-E**). After flower buds were cultured for 152 h, most PMCs were at metaphase I (27.0%; **Figure 1F**). We observed anaphase I after 160 h of culture (22.5%; **Figure 1G**). Telophase I (37.5%; **Figure 1H**) was detected under light microscopy, indicating that the first cell division had finished. PMCs took about 64 hours to complete the first division.

The second division directly started without cytokinesis. Prophase II (34.0%; **Figure 1I**) was dominant stage after 168 h of culture. Metaphase I, anaphase I, telophase I, and metaphase II, as presented in **Figure 1J**, and anaphase II, (8.0%; **Figure 1K**) were also observed. The dominant meiotic stage changed from metaphase II (37.0%; **Figure 1J**) to telophase II (50.5%; **Figure 1L**) from 174 to186 h of culture. Tetrads (81.0%) became the dominant stage with the formation of cell plates after 192 h of culture (**Figure 1M**). Few triads were observed (**Figure 1N**). The second division was completed in 32 h.

### GA_3_ injection causes defects in meiotic cytokinesis of PMCs by interfering with RMA formation at telophase II

**Figure 2:**
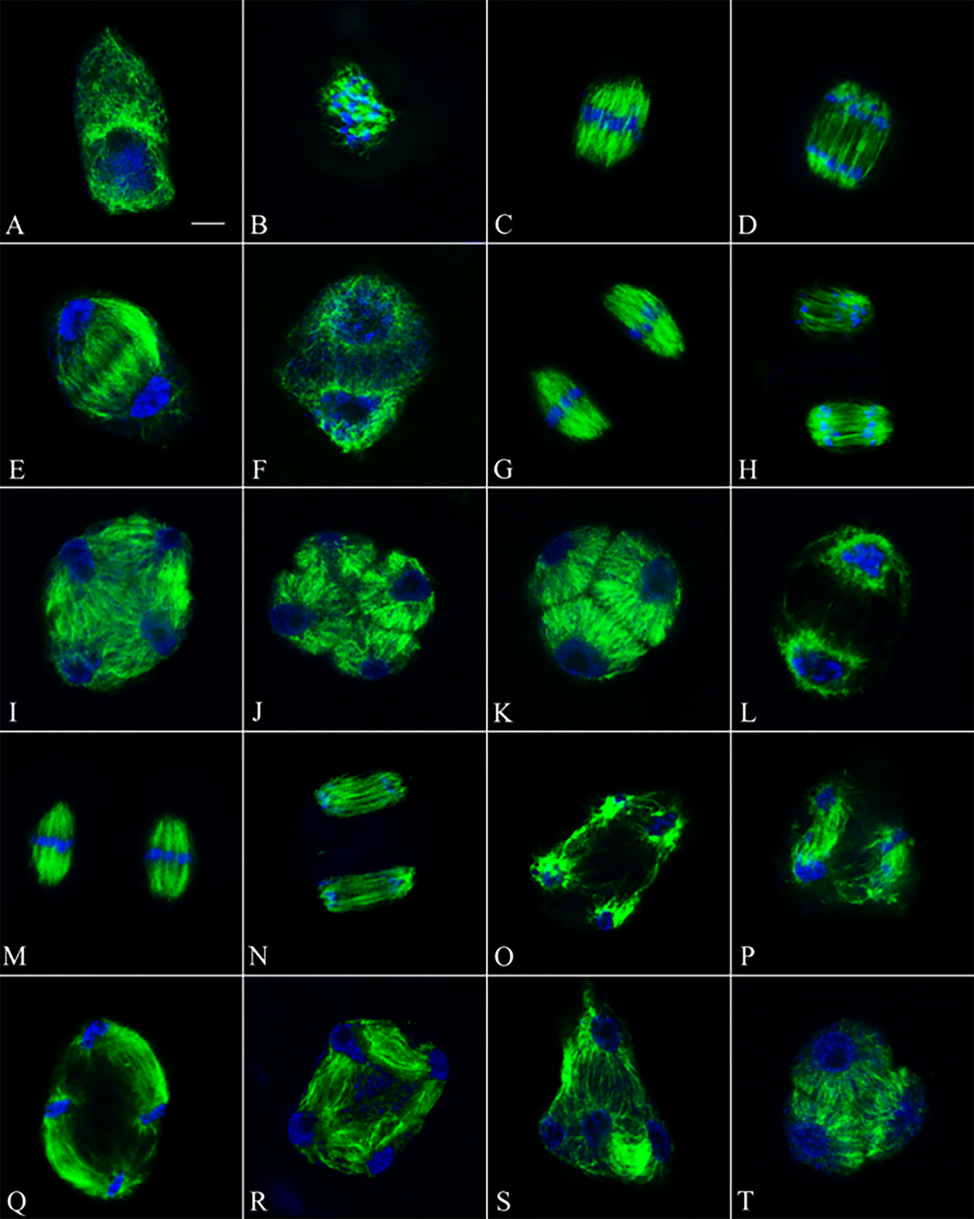
Meiotic microtubule (green) and chromosome (blue) arrangements during male meiosis in control and GA_3_-treated PMCs. **(A-K)** Microtubule and chromosome arrangements during meiosis in the control PMCs. **(A)** Leptotene in the control group. **(B)** Diakinesis in the control group. **(C)** Metaphase I in the control group. **(D)** Anaphase I in the control group. **(E)** Telophase I in the control group. **(F)** Prophase II in the control group. **(G)** Metaphase II in the control group. **(H)** Anaphase II in the control group. **(I)** Telophase II in the control group. **(J)** Tetrad in the control group. **(K)** Triad in the control group. **(L-T)** Microtubule and chromosome arrangements during meiosis in GA_3_-treated treatments. **(L)** Prophase II in the 10 μM GA_3_ treatment. **(M)** Metaphase II in the 10 μM GA_3_ treatment. **(N)** Anaphase II in the 10 μM GA_3_ treatment. **(O–S)** Defects in RMA formation of GA_3_-treated PMCs at telophase II. **(T)** Triad in the 10 μM GA_3_ treatment. Bars, 10 μm.

The phragmoplast plays an important role in the cell wall formation and deposition of cell wall components, such as intermediate callose, in somatic plant cells (De Storme and Geelen, 2013a). Microtubular phragmoplast-like structures are also necessary for cytokinesis during the male meiotic process. Nevertheless, they are referred to as RMAs due to their specific spatial intercellular organization of forming at the intersection of microtubules (MTs) emanating from syncytial telophase II nuclei (Peirson et al., 1997; Brown and Lemmon, 2001). Owing to the importance of RMAs in the formation and positioning of the meiotic cell wall (Otegui and Staehelin, 2004), we conducted immunocytological analysis of the MT subunit tubulin-α in the control and GA_3_-treated PMCs (**Figure 2**). PMCs at leptotene, in the control group, have a nucleus consisted on chromatin and a nucleolus; MTs were detected in the perinuclear region (**Figure 2A**). Subsequently,the chromatin condensed into chromosomes, while some MTs entered the nucleus; all MTs extended throughout the nucleus at diakinesis (**Figure 2B**). The chromosomes were positioned at the spindle equator at metaphase I (**Figure 2C**), and then the homologs moved to the spindle poles at anaphase I (**Figure 2D**). The MTs at telophase I, extending throughout the cytoplasm (**Figure 2E**). Immediately, chromatin condensed into chromosomes again, and at prophase II the MTs entered the two daughter nuclei (**Figure 2F**). The chromosomes were at the spindle equator at metaphase II, (**Figure 2G**). Sister chromatids separated and moved to the spindle poles of the two daughter nuclei during anaphase II (**Figure 2H**). Four daughter nuclei were produced, and RMAs widely arranged throughout the cytoplasm at telophase II (**Figure 2I**). Four microspores were released at the tetrad stage following meiotic cytokinesis (**Figure 2J**), and a few triads were visible (**Figure 2K**).

After the PMCs were treated with GA_3_, their MTs were similar to those of the control group at prophase II (**Figure 2L**), metaphase II (**Figure 2M**), and anaphase II (**Figure 2N**). However, some distinct RMAs between each of the four haploid nuclei were observed at telophase II (**Figures 2P–S**), which was not observed in the control group, suggesting that the GA_3_ injection changed the biogenesis and organization of the RMAs. In some cases, the RMAs did not form or formed partially in some of the treated PMCs (**Figures 2O–Q**). Moreover, some GA_3_-treated tetrads with regularly separated nuclei exhibited low inter-nuclear MT labeling, indicating minor aberrations in RMA biogenesis or MT stability (**Figures 2R, S**). A number of triads was seen at tetrad stage (**Figure 2T**).

**Figure 3:**
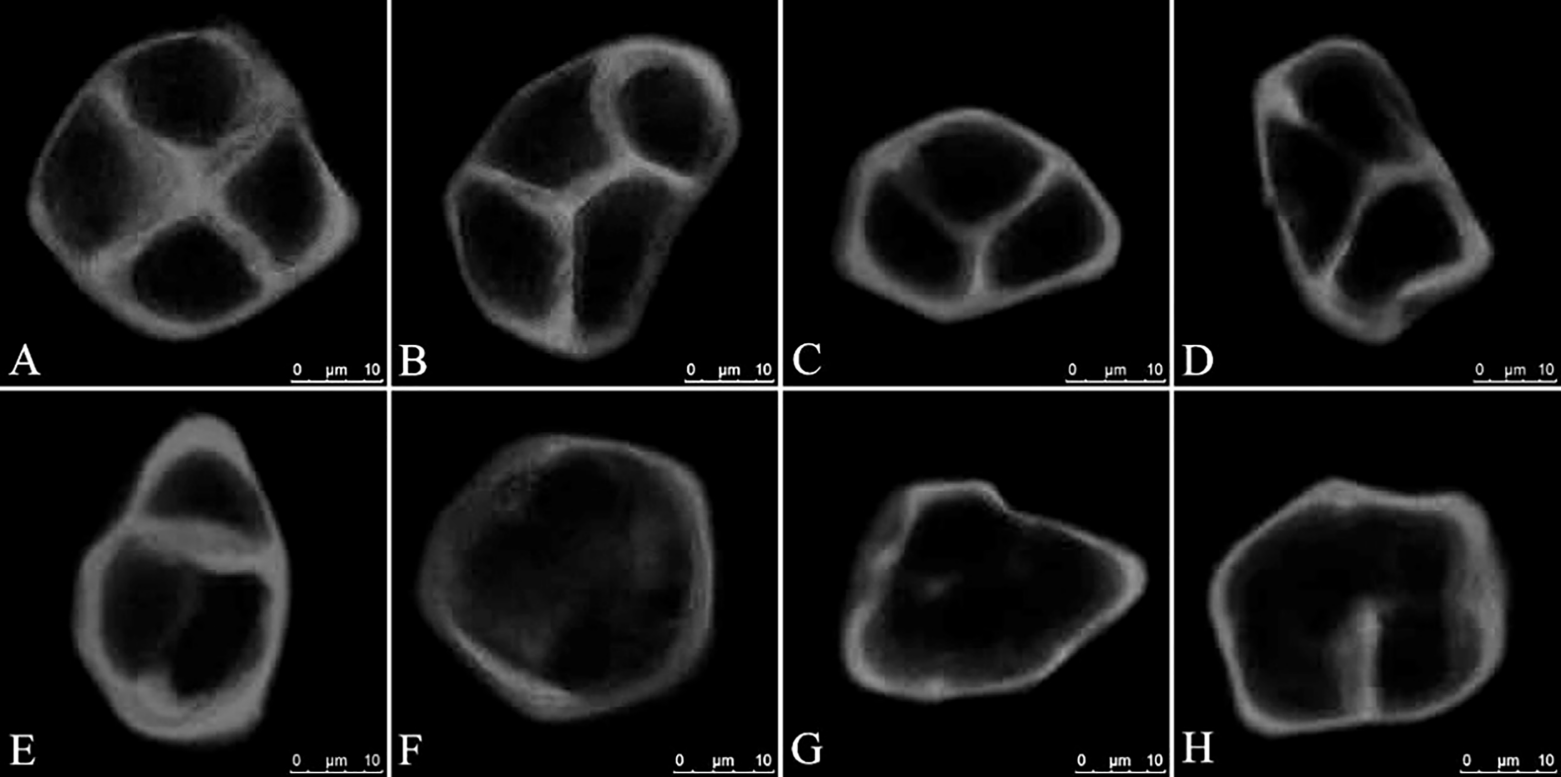
Defects in male meiotic cytokinesis in GA_3_-treated *P. bolleana* PMCs. **(A)** Aniline blue staining of tetrads in the control. **(B)** Aniline blue staining of tetrads in the GA_3_ treatment. **(C-H)** Aniline blue staining of defective tetrad stage male meiocytes in the GA_3_ treatment. Bars, 10 μm.

The accumulation of intermediate callosic material at the end of meiosis II reflects the proper formation of the cell wall and cytokinesis during male meiosis in *Populus*. Aniline blue staining of GA_3_-treated PMCs revealed changes in the deposition of callosic material in the cell walls (**Figure 3**). In the control group, almost all of the PMCs produced normal tetrads (**Figure 3A**). In some instances, GA_3_-treated PMCs also produced normal tetrads (**Figure 3B**), similar to those seen in the control, typically characterized by a distinct cross-shaped callosic cell wall separating all four haploid spores. However, at the end of meiosis II, a subset of tetrad-stage meiocytes in other GA_3_-treated PMCs produced partial or incomplete callosic cell walls, resulting in the production of balanced and unbalanced dyads and triads (**Figures 3C–E**). A large variation in defect severity was observed among this pool of altered meiotic products; some cells did not form a callosic wall (**Figure 3F, G**), whereas others generated cell wall stubs or cell plates with small gaps (**Figure 3H**). Overall, our data support that meiotic restitution resulted from defective male meiotic cytokinesis caused by defects in RMA formation.

### GA_3_ injection induces *P. bolleana* to produce 2n pollen

**Figure 4:**
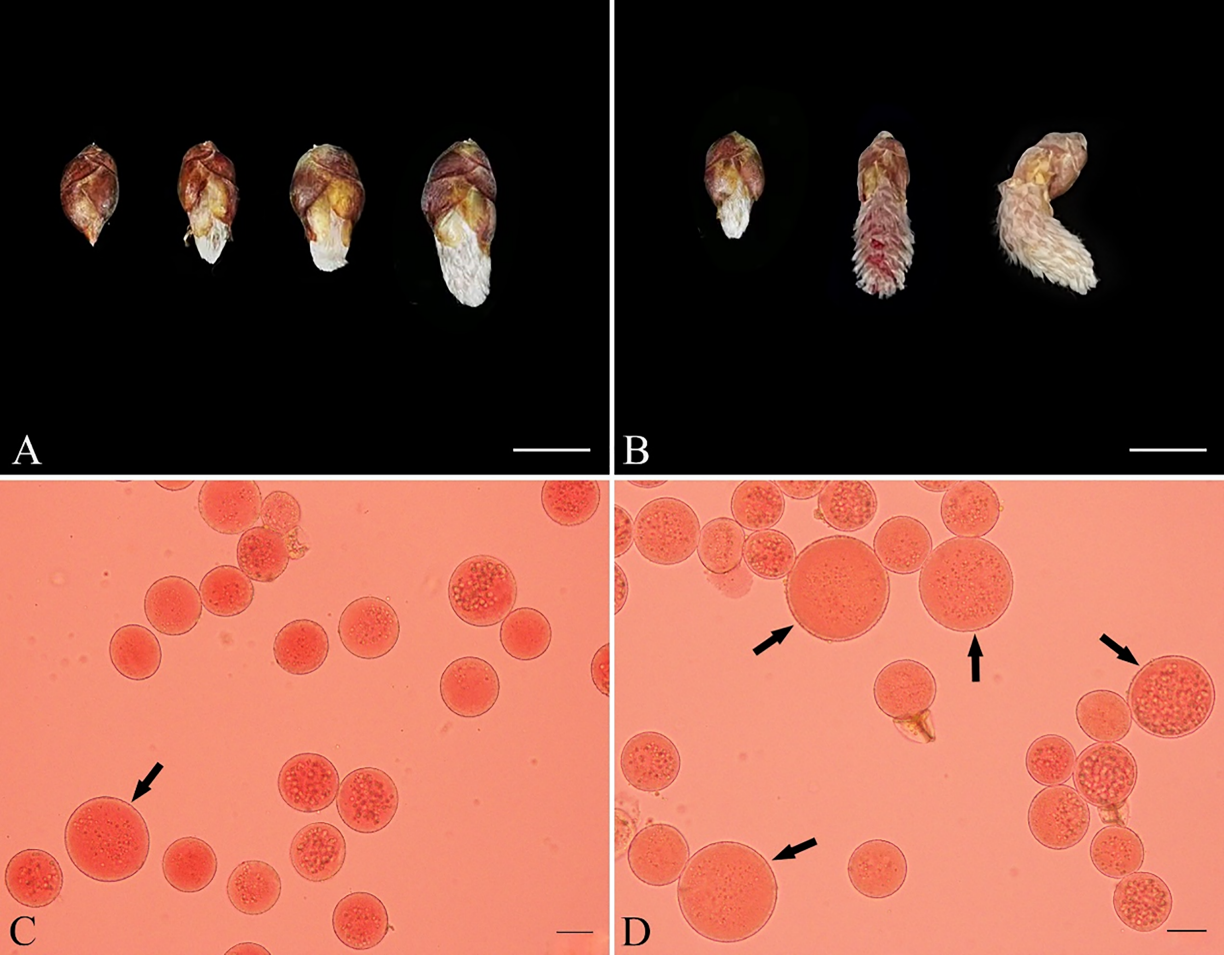
The 2n pollen induced by GA_3_ and its effects on the development of male flower buds in *P. bolleana*. **(A)** Development of flower buds on the seventh day after the GA_3_ injections. From left to right: control; flower buds given 3 injections of 10 μM GA_3_; flower buds given 5 injections of 10 μM GA_3_; flower buds given 7 injections of 10 μM GA_3_. **(B)** The dry flower buds on the seventh day after the GA_3_ injection. From left to right: control; normally developing flower buds after the 30 μM GA_3_ injection, dry flower buds after the 30 μM GA_3_ injection. Bars, 1.0 cm. **(C)** Natural 2n pollen (arrow). **(D)** Induced 2n pollen (arrow) *via* GA_3_ injection. Bars, 20 μm.

Because defects in RMA formation can be caused by GA_3_ treatment, we supposed that GA_3_ would induce male *Populus* to produce 2n pollen by interfering with male meiotic cytokinesis. To determine the effect of gibberellins on male sporogenesis in *Populus*, we injected male *P. bolleana* flower buds with 10, 30, or 50 μM GA_3_ and water, respectively. The male flower buds developed rapidly after the GA_3_ injection (**Figure 4A**), and a few anthers became dry and brown (**Figure 4B**). Some buds died after the GA_3_ treatment, resulting in no pollen.

Surviving flower buds were cultured in the greenhouse until the anthers matured. The ploidy of the pollen was analyzed after the GA_3_ treatment to assess the putative changes in male spore formation. The ploidy level of the induced pollen grains can be determined by assessing the pollen diameter, which is a proxy for the ploidy level of the gametophyte (De Storme and Geelen, 2013a). In the control group, a few spontaneous 2n pollen grains were detected (**Figure 4C**). The frequency of natural 2n pollen was 0.48%. GA_3_-induced 2n pollen was collected from all surviving treated male buds (**Figure 4D**). The frequencies of GA_3_-induced 2n pollen dervied from different treatments are showed in **Table 2**. The average frequency of GA_3_-induced 2n pollen changed from 1.89% to 21.37%. A general linear model (GLM)-univariate analysis of the frequencies of GA_3_-induced 2n pollen indicated that the dominant meiotic stage (F = 41.882, *P* < 0.001), GA_3_ injection time (F = 172.466, *P* < 0.001) and dominant meiotic stage × GA_3_ concentration interaction (F = 13.642, *P* < 0.001) significantly affected the frequency of induced 2n pollen. However, the GA_3_ concentration (F = 1.391, *P* = 0.253) had no significant effect on the frequency of induced 2n pollen. Subsequently, the least significant difference multiple-comparison tests revealed that significantly more 2n pollen was induced at prophase II than that at other stages (*P* < 0.05). The frequency of induced 2n pollen was significantly higher in samples given 7 injections than in samples given 3 or 5 injections. Therefore, the optimal combination for inducing 2n pollen by GA_3_ was to give 7 injections of 10 μM GA_3_ at the prophase II stage of the PMCs.

**Table 2:** Induction of 2n pollen *via* GA_3_ injection in *P. bolleana*.

Dominant meiotic stage of PMCs	Concentration of GA_3_ (μM)	No. of GA_3_ injection times	Frequencies of induced 2n pollen (%)
Diakinesis	10	3	1.89 ± 0.20
		5	2.57 ± 0.55
		7	6.66 ± 1.16
	30	3	3.36 ± 1.68
		5	4.71 ± 0.68
		7	5.51 ± 1.40
	50	3	3.29 ± 1.21
		5	5.92 ± 2.37
		7	11.13 ± 0.80
Metaphase I	10	3	8.53 ± 2.97
		5	6.01 ± 2.39
		7	9.56 ± 1.48
	30	3	4.58 ± 2.49
		5	5.79 ± 1.92
		7	13.67 ± 4.91
	50	3	2.63 ± 1.06
		5	3.96 ± 1.23
		7	12.19 ± 2.88
Anaphase I	10	3	7.22 ± 0.96
		5	12.37 ± 2.46
		7	14.84 ± 0.81
	30	3	7.04 ± 1.19
		5	10.40 ± 1.20
		7	10.80 ± 1.16
	50	3	4.02 ± 0.38
		5	5.66 ± 1.31
		7	12.56 ± 1.60
Prophase II	10	3	6.87 ± 1.21
		5	15.65 ± 1.02
		7	21.37 ± 1.89
	30	3	7.39 ± 1.83
		5	8.06 ± 0.98
		7	12.52 ± 0.64
	50	3	5.02 ± 0.54
		5	6.33 ± 1.00
		7	19.99 ± 1.86
Metaphase II	10	3	5.48 ± 1.64
		5	12.03 ± 2.16
		7	6.12 ± 1.45
	30	3	11.84 ± 1.07
		5	11.98 ± 2.21
		7	13.75 ± 1.82
	50	3	4.37 ± 0.96
		5	10.00 ± 0.28
		7	11.34 ± 2.79
Anaphase II	10	3	2.17 ± 0.31
		5	13.41 ± 2.33
		7	6.95 ± 1.66
	30	3	6.67 ± 1.66
		5	15.24 ± 1.75
		7	6.43 ± 2.07
	50	3	5.87 ± 1.22
		5	9.54 ± 1.84
		7	16.96 ± 3.45
Prophase II (Control)	0	5	0.48 ± 0.05

### Inducing 2n pollen *via* a GA_3_ injection in other *populus* species

**Figure 5:**
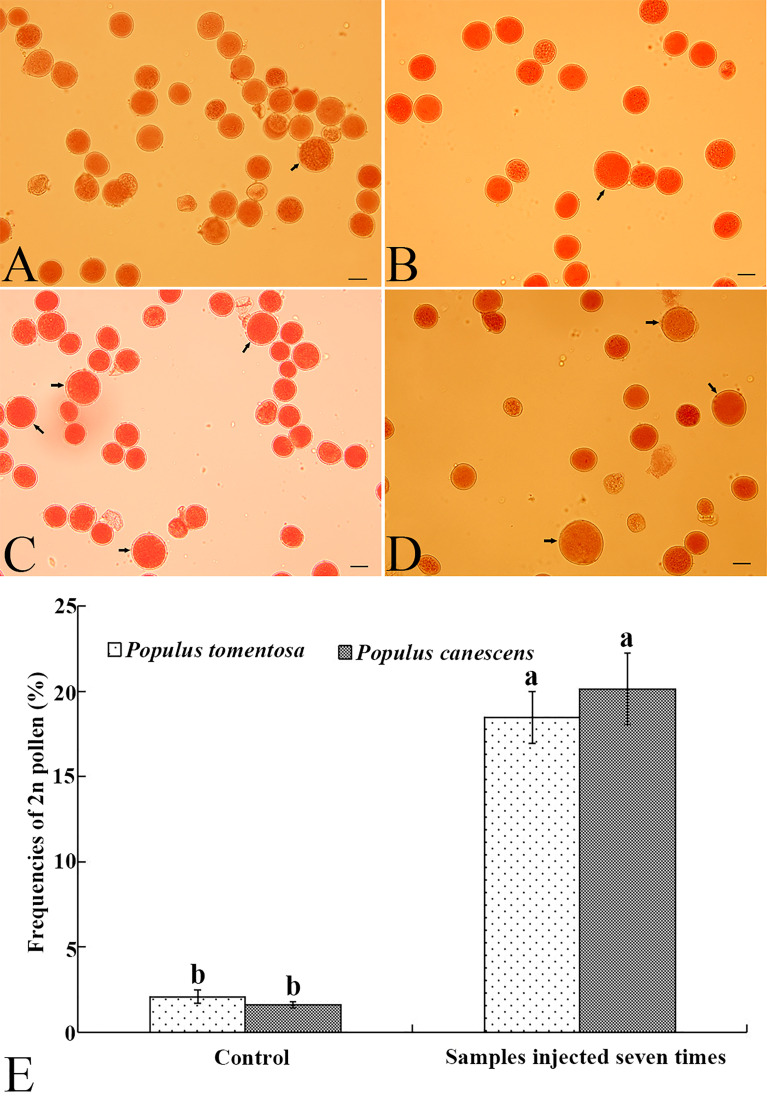
Natural 2n pollen, induced 2n pollen *via* a GA_3_ injection, and frequencies of 2n pollen in *P. canescens* and *P. tomentosa* clone 5088. **(A)** Natural 2n pollen in *P. canescens* (arrow). **(B)** Natural 2n pollen in *P. tomentosa* clone 5088 (arrow). **(C)** GA_3_-induced 2n pollen in *P. canescens* (arrow). **(D)** GA_3_-induced 2n pollen in *P. tomentosa* clone 5088 (arrow). **(E)** The frequencies of GA_3_-induced 2n pollen in *P. canescens* and *P. tomentosa* clone 5088 after male buds were given 7 injections of 10 μM GA_3_. Lowercase letters show significant differences at the *P* < 0.05 level (*t*-test). Bars in A–D, 20.0 μm.

To test the feasibility of inducing 2n pollen with GA_3_, we subsequently treated male flower buds of *P. canescens* and *P. tomentosa* clone 5088 with 10 μM GA_3_ when most of the PMCs reached prophase II. After the male flower buds were given 7 injections, all treated male branches continued to be cultured in the greenhouse until the pollen was released from the anthers. Similar to *P. bolleana*, a few natural 2n pollen grains were detected in the *P. canescens* (**Figure 5A**) and *P. tomentosa* clone 5088 (**Figure 5B**) control groups. The frequencies of natural 2n pollen produced were 2.08% and 0.48%, respectively. Among the treated groups, GA_3_-induced 2n pollen grains were observed in *P. canescens* (**Figure 5C**) and *P. tomentosa* clone 5088 (**Figure 5D**). The frequencies of GA_3_-induced 2n pollen were 18.46% and 20.13%, respectively which were significantly higher than those of the control groups (**Figure 5E**).

### Triploid production by crossing GA_3_-induced 2n pollen

**Figure 6:**
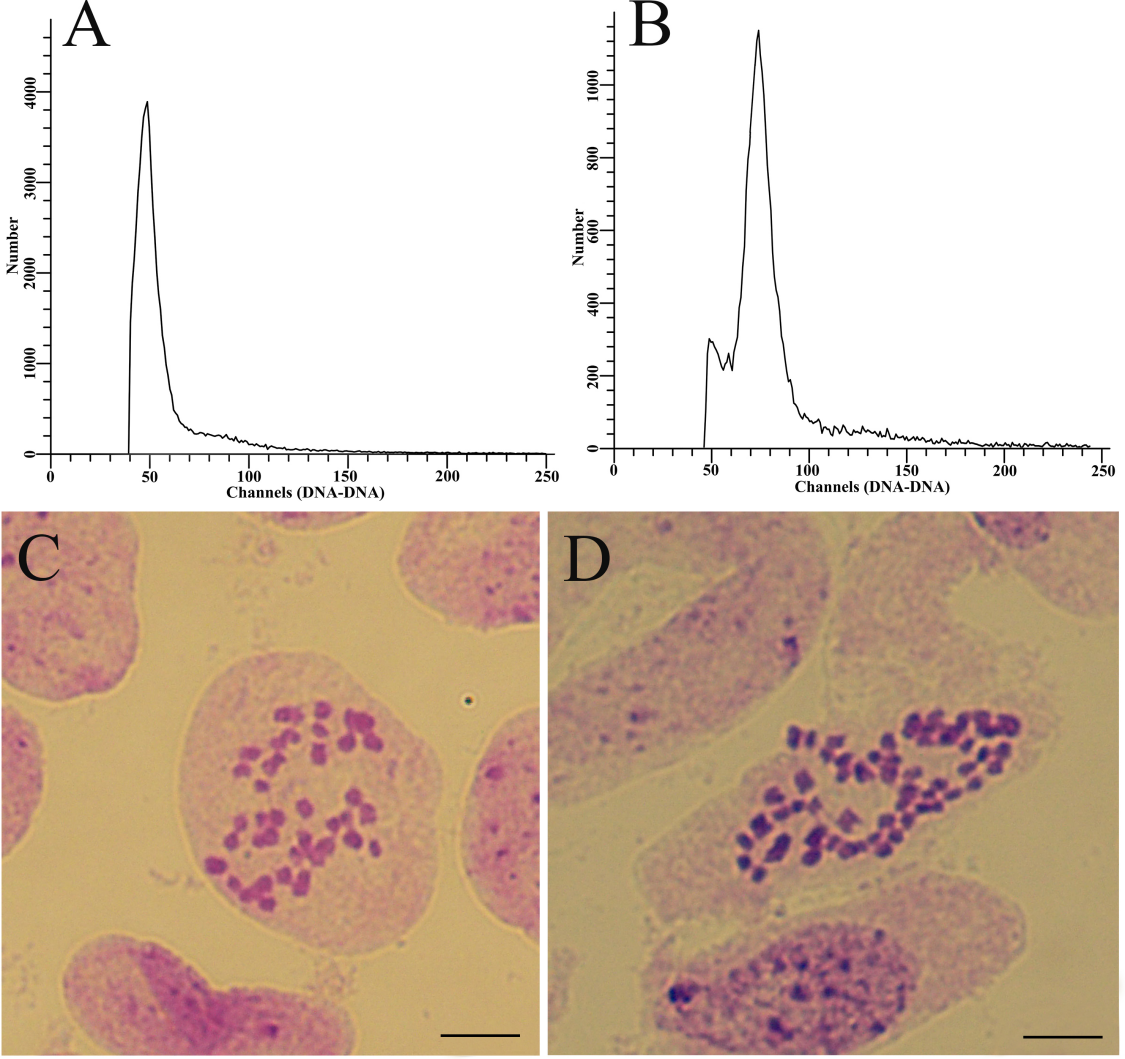
Ploidy level analysis of offspring derived from female gametes in *P. alba × P. glandulosa* crossing GA_3_-induced 2n pollen in *P. bolleana*. **(A)** Flow cytometry of diploid plants. **(B)** Flow cytometry of triploid plants. **(C)** Chromosome number of diploid plants (2n = 2x = 38). **(D)** Chromosome number of triploid plants (2n = 2x = 57). Bars, 5.0 μm.

According to **Table 2**, ten combinations of treatments with high frequencies of induced 2n pollen in *P.bolleana* were selected for crossed with female gametes in *P.alba× P.glandulosa.* Meanwhile, the GA_3_-induced 2n pollen in *P.canescens* obtained after the optimal combination of treatments was crossed with female gametes in *P.alba × P.glandulosa.* A total of 4,896 seeds were obtained from cross combinations with different frequencies of GA_3_-induced 2n pollen and the control groups. 3381 seeds in total were sown and developed into young seedlings (**Table 3**). According to the flow cytometry peaks, individual seedlings were divided into diploid (**Figure 6A**) or triploid (**Figure 6B**). Finally, 18 putative triploids were detected. The chromosome number in diploids was 38 (**Figure 6C**), and all putative triploids were confirmed to be real triploids (2n = 2x = 57, **Figure 6D**). All triploids were produced from these offspring with different GA_3_-induced 2n pollen. No triploids were detected in the control groups, indicating that *P. alba × P. glandulosa* did not generate 2n eggs, and no natural 2n pollen participated the fertilization of the control group.

**Table 3:** Crossing GA_3_-induced 2n pollen with female gametes in *P. alba × P. glandulosa* to produce triploids.

Cross combinations	Frequencies of induced 2n pollen (%)	Number of seeds	Number of seedlings	Number of triploids
(*P. alba × P. glandulosa*) *× P. bolleana*	5.92	22	17	1
	9.56	172	145	0
	12.37	370	224	1
	14.84	46	40	1
	9.33	107	78	1
	25.17	1112	922	5
	19.99	421	270	0
	13.75	126	97	2
	15.24	420	342	3
	16.96	47	38	0
	0.48 (control)	300	263	0
(*P. alba × P. glandulosa*) *× P. canescens*	18.46	786	742	4
	2.08 (control)	967	703	0
	Total	4896	3881	18

## Discussion

We revealed an unexpected function of GAs using a combination of tubulin-α immunolocalization and pollen chromosome doubling technology. In particular, injecting male flower buds of *Populus* with exogenous GA_3_ caused defects in male meiotic cytokinesis by interfering with RMA formation, leading to meiotic restitution and 2n pollen production. Subsequently, we used GA_3_ to treat the male flower buds at different meiotic stages, and a large number of induced 2n pollen grains was produced by *P. bolleana*, *P. canescens*, and *P. tomentosa* clone 5088. Eighteen triploids were generated by crossing haploid female gametes of *P. alba × P. glandulosa* with GA_3_-induced 2n pollen of *P. bolleana* and *P. canescens.* Hence, GA_3_ could be used as a novel chemical mutagen to induce 2n pollen production.

Various mechanisms contribute to the origin of polyploid species in plant kingdom (Ramsey and Schemske, 1998). However, the production of unreduced gamete is thought to the main cause of induced polyploid (Bretagnolle and Thompson, 1995). Both pre- and post-meiotic genome doubling events, as well as meiotic restitution, generally cause the 2n gamete formation (Bretagnolle and Thompson, 1995; Ramsey and Schemske, 1998). For example, ectopic defects in the formation of cell wall during mitotic cell division in meiotic founder cells cause premeiotic genome doubling and the associated formation of tetraploid meiocytes and diploid pollen grains were reported in the tomato *pmcd1* mutant (De Storme and Geelen, 2013b). According to the main cellular process driving sexual polyploidization, meiotic restitution has been divided into three mechanisms: (1) omission of meiosis I or II, (2) changes in spindle organization, and (3) unsuccessful or incomplete meiotic cytokinesis (Ramanna and Jacobsen, 2003).

Defects in meiotic cytokinesis can lead to meiotic restitution, either following meiosis I or II, with either full or partial elimination of the meiotic cell wall causing the 2n or polyploid gamete formation (De Storme and Geelen, 2013c). A specific MAPK signaling cascade regulates male meiotic cytokinesis in Arabidopsis (Takahashi et al., 2004; Takahashi et al., 2010). Lack of function of TES, MKK6, or MPK4 leads to the defective formation of the male meiotic cell wall resulting in ectopic diploid or polyploid male gamete production (Spielman et al., 1997; Kosetsu et al., 2010; Zeng et al., 2011). Liu et al. (2017) reported that defective male meiotic cytokinesis were observed when flowering Arabidopsis is sprayed with exogenous GA_3_ (100 μM) and less than 5.0% of 2n pollen is produced. In the present study, we injected male *P. bolleana* buds with 10 μM GA_3_ when the dominant meiotic stage of the PMCs was prophase II, and more than 20.0% 2n pollen was generated. Some defects in male meiotic cytokinesis resulting from defects in RMA formation were seen, suggesting that the exogenous GA_3_ treatment can interfere with RMA formation leading to defects in male meiotic cytokinesis of *Populus*.

Colchicine is a mutagen used to induce 2n gamete production in plants because it can prevent from microtubule polymerization though binding to tubulin during cell division (Mashkina et al., 1989). Colchicine has been widely used to induce 2n gametes, and a lot of triploids were generated in *Populus*. For example, Kang et al. (2004) recorded that the highest frequency of colchicine-induced 2n pollen in *P. tomentosa* × *P. bolleana* was 88.0% in a white *Populus* polyploid breeding program, and 12 triploids were obtained by crossing a high frequency (63.7%) of induced 2n pollen. Kang et al. (2004) obtained 21 triploids by doubling the embryo sac number of chromosomes using colchicine. Li et al. (2008) produced 12 triploids by doubling the number of megaspore chromosomes with colchicine. However, the use of colchicine has been restricted in some countries owing to its high toxicity. Non-toxic GA_3_ is a much safer mutagenic agent for inducing 2n gamete production in plants.

Applying a mutagenic agent to cells that are at a suitable meiotic stage is important for inducing chromosome doubling during gametogenesis. Former studies have shown that the pachytene stage of meiosis is optimal for producing colchicine-induced 2n pollen of *P. tomentosa* × *P. bolleana* (Kang et al., 1999) and inducing 2n megaspores in *P. alba* × *P. glandulosa* (Li et al., 2008). Tian et al. (2018) revealed that diakinesis was the most suitable meiotic stage for high-temperature induction of 2n pollen during microsporogenesis of *P. canescens*. However, in the present study, prophase II of the PMCs was the most accurate meiotic stage for 2n pollen induction by GA_3_ in *P. bolleana*, which is much later than the optimal meiotic stages induced by colchicine and high-temperature exposure. These results suggest that exogenous GA_3_ interferes with RMA formation, and it takes time for exogenous GA_3_ to take function in PMCs.

The injection time of the chemical mutagen is vital when inducing gamete chromosome doubling at the appropriate developmental stage of cells in *Populus*. In former studies, the frequency of colchicine induced 2n pollen differed significantly from colchicine injection times in *P. tomentosa* × *P. bolleana* (Kang et al., 1999), *P. alba* (Li et al., 2014), *P.* × *popularis* (Xi et al., 2011), and *P. canescens* (Zhou et al., 2020). The frequency of colchicine-induced 2n pollen increased as the number of injections was increased. Increasing the number of injection times yielded increased frequency of induced 2n pollen up to a point, as induced 2n pollen production per catkin decreased sharply when the number of injections was excessive. Thus, the suitable number of times to inject colchicine is critical to produce a large number of induced pollen grains. In the present study, the number of GA_3_ injections had a significant effect on the frequency of induced 2n pollen, and the frequency of induced 2n pollen in samples injected 7 times was significantly higher than those injected 3 or 5 times. After male flower buds were treated with GA_3_, the pollen production per catkin also decreased (data not shown).

## Data availability statement

The raw data supporting the conclusions of this article will be made available by the authors, without undue reservation.

## Author contributions

PZ designed the research. YZ, KB, PD, LL, JD, LM, YS, JW, QZ, and XC performed the research. YZ analyzed the data and wrote the original draft. PZ and XK reviewed and edited the paper. All authors contributed to the article and approved the submitted version.
